# The concordance between the volume hotspot and the grade hotspot: a 3-D reconstructive model using the pathology outputs from the PROMIS trial

**DOI:** 10.1038/pcan.2016.7

**Published:** 2016-07-12

**Authors:** A El-Shater Bosaily, M Valerio, Y Hu, A Freeman, C Jameson, L Brown, R Kaplan, R G Hindley, D Barratt, M Emberton, H U Ahmed

**Affiliations:** 1 Division of Surgery and Interventional Science, University College London, London, UK; 2 Department of Urology, University College London Hospitals NHS Foundation Trust, London, UK; 3 Department of Urology, Centre Hospitalier Universitaire Vaudois, Lausanne, Switzerland; 4 Centre for Medical Image Computing, University College London, London, UK; 5 Department of Histopathology, College London Hospitals NHS Foundation Trust, London, UK; 6 Medical Research Council Clinical Trials Unit, University College London, London, UK; 7 Department of Urology, Basingstoke Hospital, Hampshire Hospitals NHS Foundation Trust, Hampshire, UK

## Abstract

**Objectives:**

The rationale for directing targeted biopsy towards the centre of lesions has been questioned in light of prostate cancer grade heterogeneity. In this study, we assess the assumption that the maximum cancer Gleason grade (Gleason grade hotspot) lies within the maximum dimension (volume hotspot) of a prostate cancer lesion.

**Methods:**

3-D histopathological models were reconstructed using the outputs of the 5-mm transperineal mapping (TPM) biopsies used as the reference test in the pilot phase of Prostate Mri Imaging Study (PROMIS), a paired validating cohort study investigating the performance of multi-parametric magnetic resonance imaging (MRI) against transrectal ultrasound (TRUS) biopsies. The prostate was fully sampled with 5 mm intervals; each core was separately labelled, inked and orientated in space to register 3-D cancer lesions location. The data from the histopathology results were used to create a 3-D interpolated reconstruction of each lesion and identify the spatial coordinates of the largest dimension (volume hot spot) and highest Gleason grade (Gleason grade hotspot) and assess their concordance.

**Results:**

Ninety-four men, with median age 62 years (interquartile range, IQR= 58–68) and median PSA 6.5 ng ml^−1^ (4.6–8.8), had a median of 80 (I69–89) cores each with a median of 4.5 positive cores (0–12). In the primary analysis, the prevalence of homogeneous lesions was 148 (76% 95% confidence interval (CI) ±6.0%). In all, 184 (94±3.2%) lesions showed concordant hotspots and 11/47 (23±12.1%) of heterogeneous lesions showed discordant hotspots. The median 3-D distance between discordant hotspots was 12.8 mm (9.9–15.5). These figures remained stable on secondary analyses using alternative reconstructive assumptions. Limitations include a certain degree of error within reconstructed models.

**Conclusions:**

Guiding one biopsy needle to the maximum cancer diameter would lead to correct Gleason grade attribution in 94% of all lesions and 79% of heterogeneous ones if a true hit was obtained. Further correlation of histological lesions, their MRI appearance and the detectability of these hotspots on MRI will be undertaken once PROMIS results are released.

## Introduction

Correct risk attribution is of key importance to guide appropriate management for men with prostate cancer. Our current diagnostic pathway based on transrectal ultrasound (TRUS) guided biopsy can result in inaccurate risk stratification in up to half of all men diagnosed.^1^ This can then result in missed diagnoses and under-treatment as well as the more commonly recognised issues of over-diagnosis and over-treatment.^2, 3, 4, 5, 6^

Recent research has focused on using multi-parametric magnetic resonance imaging (mpMRI) to improve the diagnostic accuracy by introducing tumour location at the time of biopsy.^7, 8, 9^ The provision of information on tumour location means that biopsies can be directed to the region of interest rather than be solely spread across the prostate as currently done with random TRUS biopsies. Some have argued that the ‘targeted approach' might result in employing fewer needle deployments than we have previously used.^10, 11^ If this is to happen—and it would be desirable if it could—then we would need to know where within the ‘target' to direct those needles.

It is customary when presented with a target to direct the needle to the centre of the target. However, some have raised concerns that this strategy may not be optimal.^12, 13^ Instead, it has been argued that information acquired from imaging can identify a particular area to target in order to obtain the most aggressive component of one lesion which may not be.

To further explore this question, we have analysed data obtained from the pilot phase of the MRC (Medical Research Council)/HTA (Health Technology Assessment) Prostate Mri Imaging Study (PROMIS), in which biopsy-naive men underwent mpMRI followed by a transperineal template prostate mapping (TPM) biopsy and TRUS biopsy. In this study, we attempted to assess the validity of the premise that the largest dimension of a tumour (volume hotspot) harbours the highest Gleason grade (Gleason grade hotspot).

## Materials and methods

### Design of the PROMIS trial

PROMIS is a multicentre paired validating cohort study (Trial registry identifiers: ISRCTN16082556, NCT01292291) funded by the UK NIHR (National Institute of Health Research)—HTA programme and designed to assess the diagnostic accuracy of an mpMRI-dependent pathway in detection of clinically significant prostate cancer.^14^

**Figure 1:**
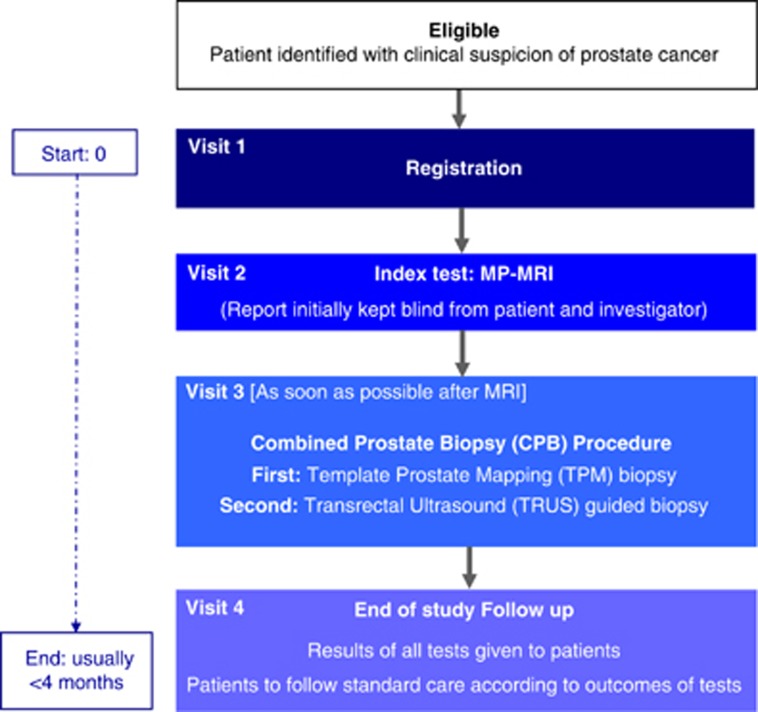
Prostate Mri Imaging Study (PROMIS) trial schema. MP-MRI, multi-parametric magnetic resonance imaging.

Biopsy-naive men who have been recommended a biopsy for suspicion of prostate cancer (elevated PSA, abnormal digital rectal examination, family history and/or ethnic risk group) are offered participation to the trial (Table 1). After an informed consent is obtained, patients undergo a standardised mpMRI (index test) protocol compliant to the European Society of Urogenital Radiology guidelines^15^ followed by a combined biopsy procedure involving TPM biopsy (reference test) and TRUS biopsy (standard test) under general anaesthetic (Figure 1). MpMRI results are blinded to clinicians performing the biopsies and to pathologists reporting the biopsy results.

For the purpose of this work, we have used the histological outputs from TPM biopsies conducted within the pilot phase of the trial. The pilot phase was unique in permitting such an analysis as each individual core was potted, processed and reported separately as well as oriented in space (cranio-caudal and x-y plane). The full trial incorporated 5 mm sampling but potted a number of cores together within prostate zones so would not permit such an analysis as this. Imaging results and its correlation to histology form the primary objective of the trial, we do not make any reference to imaging findings within this paper as our aim is to asses the validity of the premise that the highest Gleason score resides in the lesion's largest dimension, not if is consistent with a specific, identifiable imaging phenotype.

### TPM biopsy

TPM biopsy has been chosen as the reference standard to validate mpMRI in PROMIS. It produces a histological map of the entire prostate in 3-dimensions with an estimated sensitivity and negative predictive value of around 95% (relative to prostatectomy) for clinically significant cancer.^16, 17^

In TPM, the prostate is sampled every 5 mm. This procedure has been described in detail elsewhere.^18^ Only within the pilot phase of PROMIS, each biopsy core was individually 3-D oriented in space in concordance to its location in the prostate by recording the brachytherapy template grid (‘x' and ‘y' planes) coordinates whilst the apical end of each core was stained with India ink to identify its cranio-caudal orientation (‘z' plane).

**Figure 2:**
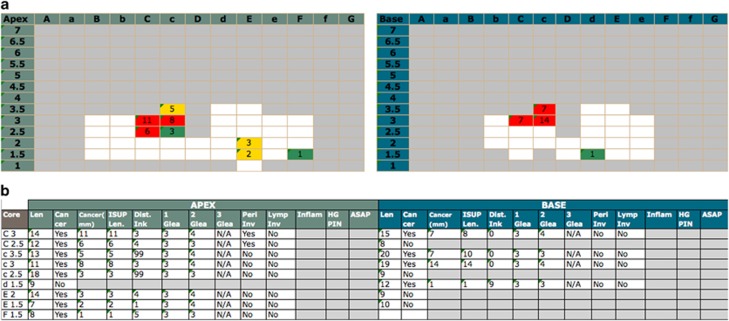
Transperineal mapping (TPM) histological findings per patient within the Prostate Mri Imaging Study (PROMIS) trial are resumed by a full report and by a visual report. In the full report (**a**), each core is labelled per coordinate and the following details are displayed: core length, cancer status, cumulative and separate cancer core length (CCL), cancer position, primary, secondary and tertiary Gleason grade, perineural and lymphovascular invasion as well as the presence of inflammation, high-grade PIN and ASAP. The TPM visual report (**b**) provides immediate zonal location within the gland. Maximum CCL and colour-coded risk attribution are displayed per coordinate with white boxes representing prostate biopsies with no cancer. ASAP, atypical small acinar proliferation; PIN, prostatic intraepithelial neoplasia.

**Figure 3:**
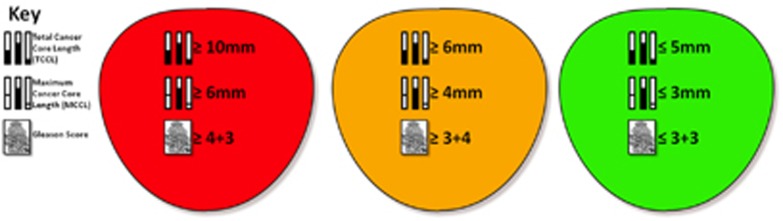
UCL definitions of clinical significance on transperineal mapping (TPM) biopsy. Red indicates UCL definition 1 for significant disease (maximum cancer core length (MCCL)⩾6mm and/or Gleason score⩾4+3). Yellow indicates UCL definition 2 for significant disease (MCCL⩾4mm and/or Gleason score⩾3+4) and green defines insignificant disease (MCCL⩽3mm and Gleason score⩽3+3). UCL, University College London.

Each core was examined by an experienced uro-pathologist and reported in terms of core length, Gleason score, high-grade prostatic intraepithelial neoplasia and inflammation. The cancer core length (CCL) was reported with the distance from the apical aspect of the core. Results were plotted on a visual map with colour-coding to reflect the risk stratification derived from each core (Figures 2 and 3).^19^

This reporting format presents unprecedented detail and clarity in recording the spatial position and relationship between positive cores, their Gleason grades and estimated lesion volumes enabling us to interpolate the results into a 3-D model.

### Histological definitions

#### Volume hotspot

Volume hotspot is the coordinate in which if a biopsy needle is deployed it will sample the largest dimension of the lesion and return the longest CCL. The relationship between lesion volume and CCL is well demonstrated in Ahmed *et al.*'s^19^ previous work, and it is the basis of the lesion volume interpolation. It is determined across the sampling plane (cranio-caudal) of a template biopsy rather than on maximum dimension of the interpolated lesion, which may not be accessible from a TRUS or a template approach hence does not contribute to patient risk stratification.

#### Calculation of CCL

For the purpose of this study, decisions had to be taken to define how the CCL is calculated. There is no consensus with respect to which is the best method to define the CCL when discontinuous foci of cancer are present within the same core. Based on a recent survey around half pathologists consider that intervening benign tissue is not part of the cancer (separate count), whereas the remaining half count CCL from the initial part of the core with cancer to the end of the last cancer foci, regardless of the amount of benign tissue in between (cumulative count).^20^ In this study, we used separate counts within our primary analysis but also secondarily evaluated the impact of using the cumulative count.

#### Gleason hotspot

Gleason hotspot is the coordinate in which if a biopsy needle is deployed, it will capture the highest Gleason grade in the lesion independent of the overall lesion volume or Gleason score.

A *homogeneous lesion* is defined as a lesion compromised of only one Gleason pattern, and hence both Gleason and volume hotspots are inherently considered as concordant.

A*heterogeneous lesion* is defined as a lesion composed of more than one Gleason pattern, and hence the volume and Gleason grade hotspots may not be at the same biopsy coordinates (non-Concordance).

On reporting biopsies, primary and secondary Gleason grade are reported on the basis of relative percentage rather than on fixed quantitative thresholds. Therefore, in the case of one lesion generated by the combination of various cores with the same total Gleason scores (ex: Gleason 7) but different amounts of each grade pattern per core (ex: Gleason 3+4, 40% grade 4), it is difficult to determine whether there is or there is not total Gleason score heterogeneity. For the purpose of our primary analysis, we considered the presence of Gleason 3+4 and Gleason 4+3 in different areas of the same lesion was a criterion for heterogeneity as there is some evidence that such a differentiation matters.^21^ For secondary analyses, we also assumed heterogeneity within one lesion was present only when different total Gleason scores were present on biopsy. In other words, Gleason 4+3 and Gleason 3+4 within the same lesion were considered as homogeneous.

### 3-Dimensional model/map

**Figure 4:**
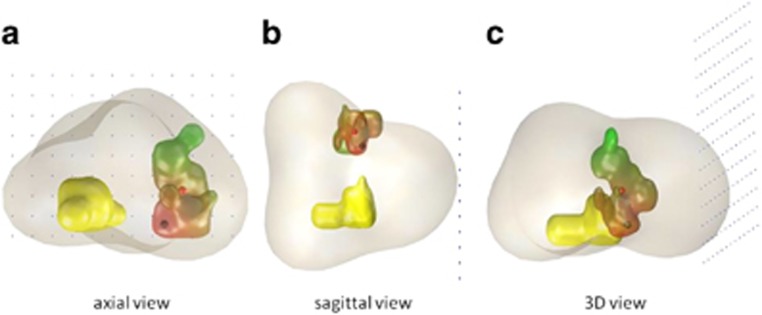
(**a**–**c**) Disease maps showing two different lesions within the same patient. One lesion (right side of the prostate; yellow) shows homogeneous grade; therefore, the hotspots are considered as concordant. The second lesion (left side of the prostate; scale of colours) shows grade heterogeneity with the Gleason grade hotspot located in the inferior right side of the lesion; therefore, the hotspots are considered as discordant.

The 3-dimensional disease maps (Figures 4a and c) were reconstructed using the detailed pathological results from all specimens. This enabled the creation of a 3-D map which potentially has 13 × 13 × 40 sections of pathological results (in terms of Gleason scores), as 13 × 13 (5 mm) template grid holes are combined with two (apex and base) needle lengths of 20 mm. Individual lesions were delineated on the reconstructed 13 × 13 × 40 map by using the rule of 26 connectivity. This means that any block of positive samples is connected to 26 potential neighbour blocks to form a single lesion. This map was then further reconstructed into a finer spatial resolution (0.5 × 0.5 × 0.5 mm^3^) by linear interpolation followed by a Gaussian smoothing, whose single parameter of isotropic variation was tuned so that the original histology results when re-sampled at the same template grid sites will be preserved. This is the simplest reconstruction algorithm from relatively sparse data maintaining the clinical validity of reconstructed lesion maps. Simulations were performed on the reconstructed map to get CCLs and Gleason scores and determine their concordance. An animation of this modelling is available at https://sites.google.com/site/yipenghu/gallery/template-biopsy-animation.

### Statistics

Descriptive statistics with continuous and categorical variables were analysed using median with interquartile range (IQR), and frequencies with percentages, respectively. Binomial 95% confidence intervals (CIs) were calculated. The data were analysed by statistical functions and procedures for descriptive statistics and significance testing, implemented in MATLAB 2014 (The MathWorks, Cambridge, UK) with Statistics Toolbox.

## Results

Overall, 129 men were enrolled in the pilot phase of PROMIS. Thirty-five were excluded for various reasons: 19 chose to withdraw; 12 because it was deemed not possible to sample the entire prostate with a 5-mm density due to gland size; 3 due to co-morbidities that developed before biopsy; and finally 1 due to accidental un-blinding of his mpMRI. Therefore, 94 were included in the present study. Patients' characteristics are reported in Table 2. Median age was 62 years (IQR= 58–68) and median PSA was 6.5 ng ml^−1^ (4.6–8.8). A median of 80 cores (69–89) were taken per patient with a median of 4.5 positive cores (0–12). Median maximum cancer core length (MCCL) was 3 mm, both when using a cumulative (0–8) and a separate (0–7) CCL count. An example of a TPM report is given in Figure 2.

Primary analyses: for our primary analyses, which used the separate criteria for defining MCCL and Gleason grades, 195 independent lesions were detected (Table 3). The overall prevalence of homogeneous lesions was 148 (95%CI 76±6.0%). Most of these lesions had a Gleason score 3+3 (*n*=119; 61±6.9%), fewer had a Gleason score 3+4 (*n*=66; 34±6.6%), and a minority had a Gleason score 4+3 (*n*=10; 5±3.1%). Median lesion volume was 0.075 ml (0.025–0.225). Discordant hotspots were present in 11/47 (23±12.1%). The median 3-D distance between the hotspots when they were discordant was 12.8 mm (9.9–15.5).

Overall, considering both homogeneous and heterogeneous lesions together, 184/195 (94±3.2%) of lesions harboured the Gleason grade hotspot in the volume hotspot.

Secondary analyses: Using separate criteria for defining the MCCL and the overall score to define heterogeneity, the results remained stable. Discordant hotspots were present in 10/43 (23±12.6%); the median 3-D distance between hotspots when they were discordant was 12.5 mm (9.9–15.8).

When the histology outputs were reconstructed to determine the 3-D models using the cumulative method to assign CCL, 190 independent lesions were found (Table 3). Most of these lesions had a Gleason score 3+3 (*n*=118; 62±6.9%), fewer had a Gleason score 3+4 (*n*=64; 34±6.7%), and a minority a Gleason score 4+3 (*n*=8; 4±2.9%). Median lesion volume was 0.075 ml (0.025–0.275). Between 144 (76±6.1%) and 148 (78±5.9%) lesions were considered as homogeneous, according to the definition of grade heterogeneity used. Of the remaining heterogeneous lesions, 33/42 (79±12.4%) and 34/46 (74±12.7%) had Gleason grade hotspots that were concordant to the volume hotspots. The median 3-D distance in the discordant lesions was 9.9 mm (9.8–15.3) and 11.5 mm (9.9–14), respectively.

The overall concordance rates of all secondary analyses when including all lesions were not different compared with the primary analysis.

## Discussion

In summary, we have shown that the Gleason grade hotspot for a lesion is concordant with the volume hotspot in over 9 in 10 of all lesions. We also found that in biopsy-naive men, about one in five lesions are heterogeneous in grade. For these lesions, the Gleason grade and volume hotspot are discordant in about 2 in 10 lesions with approximately 10 mm distance between the two.

Before discussing the clinical implications of our findings, there are some limitations that need to be addressed. First, while this is a computer reconstruction based on precise 3-D pathology data, a certain degree of error is inevitable. It is possible that some very small lesions might have been missed, and the clustering of some lesions might have been incorrect. To minimise these errors, we used two methods for determining whether positive biopsies belonged to one specific lesion or not; this had minimal impact on our findings.

Second, these findings may be valid in this study population of biopsy-naive men with early suspicion of prostate cancer and PSA less than 15 ng ml^−1^, but it is likely, and it has been indeed previously shown that greater heterogeneity is present in men with more advanced disease.^22, 23^

Third, some may argue that radical prostatectomy specimen analysis should be used as a reference test within the trial. Although we have already clarified the reasons for choosing TPM biopsy as the reference test within the PROMIS trial elsewhere,^14^ we would argue that TPM biopsy represents a more valid tool as it can avoid the selection bias towards higher disease burden associated with the use of radical prostatectomy as a reference test. This is especially true in this subgroup of biopsy-naive men, in which a minority are expected to undergo radical prostatectomy.

Finally, our study was limited to the 94 men included in the pilot phase of the trial. Although this is an embedded study with no power calculation upfront, the PROMIS TMG approved this study as these very detailed 3-D histopathological maps could be built only for this subgroup of men, and awaiting trial completion would not add additional data to this study. Indeed, while patients recruited after the pilot phase had the same TPM procedure, there was a lack of some spatial information which are of key importance for the purpose of this study (core inking and precise 3-D orientation). This change in the histopathological analysis was due to resources and cost implications.

### Clinical implications

Precise risk stratification remains a challenge. In a disease such as prostate cancer that exhibits such a degree of heterogeneity, and in which the course of the disease appears to be defined by the dominant Gleason pattern, the provision of tissue that enables its identification remains a key.^24^ The role of image-targeted biopsies has come to the fore as a way of possibly improving risk stratification. Targeted biopsies can be carried out in three possible ways: first, visually targeted, second using image-fusion software and third, in-bore (within the scanner). Recently, some have challenged the use of MRI to ultrasound registration, which currently directs the urologist's needle to the centre of a lesion by assuming that the most aggressive part of the tumour might not be in the centre of the lesion.^12^ One study showed that the most aggressive part of the prostate is not necessarily in the centre, and therefore the authors suggested targeted biopsies are best carried out in-bore. Our findings are somewhat contrasting to this report. We believe there might be some reasons for this discrepancy. First, the authors selected only men undergoing radical prostatectomy. We know that the larger the tumour the greater the heterogeneity.^22, 23^ This can be due to branching of a clone into different distinct phenotypes, but it can also be due to two adjacent, but clonally independent tumours, merging to form one.^22, 25^ Second, the authors based their results on retrospective radiological findings of diffusion coefficient heterogeneity, which does not always correspond to true histological heterogeneity.^26, 27^

Our study implies that the method of targeting and deploying a needle towards the centre of a lesion would lead to correct risk stratification in the vast majority. This reinforces the idea that MRI to ultrasound registration might be adequate to achieve correctly sampling, although the number of needles to be used to achieve a true hit in the centre of the tumour is yet to be determined taking into account that registration and operator errors can occur as well as dynamic swelling in between each needle deployment. Some early evidence from our group points to increasing accuracy of detection using up to five needle deployments per target.^28^

## Conclusion

Our study demonstrates that guiding one biopsy needle to the maximum cancer diameter would lead to correct grade attribution in the majority of all lesions and approximately 80% of lesions heterogeneous for Gleason grade. Correlation of these histological lesions to their MRI appearance as well as the optimal biopsy needle deployment protocol requires further research.

## Figures and Tables

**Table 1:** PROMIS inclusion and exclusion criteria

*Patient inclusion criteria*
Men at least 18 years or over at risk of prostate cancer who have been advised to have a prostate biopsy
Serum PSA ⩽15ng ml^−1^ within previous 3 months
Suspected stage ⩽T2 on rectal examination (organ confined)
Fit for general/spinal anaesthesia
Fit to undergo all protocol procedures including a transrectal ultrasound
Signed informed consent

*Patient exclusion criteria*
Treated using 5-alpha-reductase inhibitors at time of registration or during the prior 6 months
Previous history of prostate biopsy, prostate surgery or treatment for prostate cancer (interventions for BPH/bladder outflow obstruction are acceptable)
Evidence of a urinary tract infection or history of acute prostatitis within the last 3 months
Contraindication to MRI (e.g., claustrophobia, pacemaker, estimated GFR ⩽50)
Any other medical condition precluding procedures described in the protocol
Previous history of hip replacement surgery, metallic hip replacement or extensive pelvic orthopaedic metal work.

Abbreviations: GFR, glomerular filtration rate; MRI, magnetic resonance imaging.

**Table 2:** Patients' characteristics

*Variable*	*Value*
No. of patients	94
Age, years, median (IQR)	62 (58, 68)
PSA, ng ml^−1^, median (IQR)	6.5 (4.6, 8.8)
Total no. of cores, median (IQR)	80 (69, 89)
No. of positive cores, median (IQR)	4.5 (0, 12)
Cancer core length (separate count), median (IQR)	3 (0, 8)
Cancer core length (cumulative count), median (IQR)	3 (0, 7)

Abbreviation: IQR, interquartile range.

**Table 3:** Results of analysis after interpolation

*Variable*	*Primary analysis*	*Secondary analysis*		
*Definitions of CCL and heterogeneity*	*Separate count/Gleason grades*	*Separate count/Gleason score*	*Cumulative count/Gleason grades*	*Cumulative count/Gleason score*
No. of independent lesions	195	195	190	190

*Gleason score, no (±95% CI)*				
3+3	119 (61±6.9%)	119 (61±6.9%)	118 (62±6.9%)	118 (62±6.9%)
3+4	66 (34±6.6%)	66 (34±6.6%)	64 (34±6.7%)	64 (34±6.7%)
4+3	10 (5±3.1%)	10 (5±3.1%)	8 (4±2.9%)	8 (4±2.9%)
*Lesion volume, ml, median*	0.075	0.075	0.075	0.075
IQR	(0.025–0.225)	(0.025–0.225)	(0.025–0.275)	(0.025–0.275)
Range	(0.025–6.200)	(0.025–6.200)	(0.025–8.275)	(0.025–8.275)
Homogeneous lesions, no. (±95% CI)	148 (76±6.0%)	152 (78±5.8%)	144 (76±6.1%)	148 (78±5.9%)
Heterogeneous lesions, no. (±95% CI)	47 (24±6.0%)	43 (22±5.8%)	46 (24±6.1%)	42 (22±5.9%)
Heterogeneous lesions with concordant hotspots, no. (±95% CI)	36/47 (77±12.1%)	33/43 (77±12.6%)	34/46 (74±12.7%)	33/42 (79±12.4%)
Heterogeneous lesions with no concordant hotspots, no. (±95% CI)	11/47 (23±12.1%)	10/43 (23±12.6%)	12/46 (26±12.7%)	9/42 (21±12.4%)
3-D hotspots distance in heterogeneous non-concordant lesions, mm, median (IQR)	12.8 (9.9–15.5)	12.5 (9.9–15.8)	11.5 (9.9–14.0)	9.9 (9.8–15.3)
Total number of concordant lesions (±95% CI)	184/195 (94±3.2%)	185/195 (95±3.1%)	178/190 (94±3.5%)	181/190 (95±3.0%)

Abbreviations: CCL, cancer core length; CI, confidence interval; IQR, interquartile range.
