# Erratum: The concordance between the volume hotspot and the grade hotspot: a 3-D reconstructive model using the pathology outputs from the PROMIS trial

**DOI:** 10.1038/pcan.2016.37

**Published:** 2016-08-09

**Authors:** A El-Shater Bosaily, M Valerio, Y Hu, A Freeman, C Jameson, L Brown, R Kaplan, R G Hindley, D Barratt, M Emberton, H U Ahmed

**Keywords:** Experimental models of disease, Clinical trials, Prostate cancer, Pathology

**Correction to:** *Prostate Cancer and Prostatic Diseases* (2016); doi:10.1038/pcan.2016.7; advance online publication 12 July 2016

Following publication, the authors noticed that the conflict of interest statement was incomplete. The omitted information appears below.

DB is the director of SmartTarget Ltd. (UK company number 09847173), a medical device company the markets prostate image fusion software.

